# Optimizing the detection of hereditary predisposition in women with epithelial ovarian cancer: nationwide implementation of the Tumor-First workflow

**DOI:** 10.1007/s10689-024-00398-9

**Published:** 2024-05-29

**Authors:** Vera M. Witjes, Dorien M. A. Hermkens, Julie E. M. Swillens, Yvonne H. C. M. Smolders, Marian J. E. Mourits, Margreet G. E. M. Ausems, Joanne A. de Hullu, Marjolijn J. L. Ligtenberg, Nicoline Hoogerbrugge

**Affiliations:** 1 Department of Human Genetics, Radboud University Medical Center, Nijmegen, The Netherlands; 2 Research Institute for Medical Innovation, Radboud University Medical Center, Nijmegen, The Netherlands; 3 IQ Health Science Department, Radboud University Medical Center, Nijmegen, The Netherlands; 4 Department of Obstetrics and Gynecology, University of Groningen, University Medical Center Groningen, Groningen, The Netherlands; 5 Division Laboratories, Pharmacy and Biomedical Genetics, Department of Genetics, University Medical Center Utrecht, Utrecht, The Netherlands; 6 Department of Obstetrics and Gynecology, Radboud University Medical Center, Nijmegen, The Netherlands; 7 Department of Pathology, Radboud University Medical Center, Nijmegen, The Netherlands

**Keywords:** Epithelial ovarian cancer, Heredity, BRCA, Innovation, Multidisciplinary, Implementation

## Abstract

Genetic testing in patients with ovarian carcinoma (OC) is crucial, as around 10–15% of these women have a genetic predisposition to OC. Although guidelines have recommended universal germline testing for all patients with OC for a decade, implementation has proved challenging, thus resulting in low germline-testing rates (around 30–50%). Many new initiatives to improve genetic-testing rates have emerged, but most have been carried out at the local level, leading to differences in workflows within and between countries. We present an example of a nationwide implementation project that has successfully led to a uniform, high-quality genetic-testing workflow for women with OC. Nationwide multidisciplinary meetings generated consensus on the preferred workflow for OC genetic testing: the “Tumor-First” workflow. This workflow means starting by testing the tumor DNA for the presence of pathogenic variants in OC-risk genes, thus providing a prescreen to germline testing while yielding information on the effectiveness of treatment with PARP inhibitors. This new workflow efficiently stratifies genetic counseling and germline testing and reduces healthcare costs. Although challenging, the nationwide implementation of this workflow was successful, resulting in tumor-DNA testing rates exceeding 80%. In this article, we present our structured implementation approach, illustrate our implementation strategies—which were tailored to identified factors important to implementation—and share the lessons learned from the Tumor-First implementation project. This knowledge could facilitate the future implementation of workflows aimed at optimizing the recognition of hereditary cancers.

**Supplementary Information:**

The online version contains supplementary material available at 10.1007/s10689-024-00398-9.

## Introduction

Clinical cancer genetics is a rapidly evolving multidisciplinary medical field, in which new evidence-based knowledge regularly leads to updates in current clinical guidelines or to the creation of new guidelines. About a decade ago, the genetic-testing guidelines for ovarian carcinoma (OC) (i.e., carcinomas of the ovary, fallopian tube and peritoneum) were altered to call for the universal germline testing of each individual patient with OC [1–3]. This recommendation was based on the high likelihood (10–15%) of detecting a genetic predisposition among women with OC [4–7], which is not predicted well by patient or family characteristics [6]. The identification of a genetic predisposition to OC (i.e., a germline-pathogenic variant [PV] in *BRCA1, BRCA2, RAD51C, RAD51D, BRIP1 and PALB2*) is crucial. In addition to providing information on the effectiveness of personalized treatment with PARP inhibitors (in case of a *BRCA1/2* PV) [8, 9], it provides relatives with the opportunity to take measures to reduce their own risk of developing (or dying from) cancer [10].

The implementation of new guidelines is known to be challenging [11]. As highlighted in a recent systematic review, international germline-testing rates were only 30%, although the rates reported in more recent studies are higher than those reported in older studies [12]. Given that testing rates fell far short of universal testing, initiatives aimed at efficiently improving the detection of heredity in OC have been implemented around the world. In many cases, however, alternative workflows are implemented at the local level, thereby leading to heterogeneity in genetic testing within a given country [4, 5, 13]. Standardization within countries is important for reducing variation in patient care and for effectively leveraging the expertise of clinical genetics to safeguard the quality of genetic testing. In this article, we share the example of the “Tumor-First” project, which led to a nationwide uniform genetic-testing workflow for OC, with high tumor testing rates (exceeding 80%), thus improving the detection of genetic predisposition.

## The need for national coordination of genetic-testing workflows

Genetic-testing practice in the Netherlands (a European country with around 18 million inhabitants) illustrates how local initiatives aimed at optimizing genetic testing for OC can generate variations in workflows within a country. The Netherlands has a well-developed healthcare system, in which everyone has mandatory basic insurance that covers a wide range of services, including oncological care and genetic testing. The country has nine centers of expertise on gynecological oncology and eight centers for clinical genetics. Since 2015, the Dutch guideline for hereditary OC has stated that all women with OC are eligible for genetic testing [3]. At that time, this meant that all women with OC should have been offered the possibility of referral to a clinical geneticist for counseling and germline testing. Implementation of this workflow was nevertheless disappointing. According to a recent study, prior to intervention, fewer than 50% of women with OC underwent germline testing (and only 56% had been offered germline testing) [14].

Two alternatives to this regular genetic-testing workflow were initiated locally in the Netherlands. The first involved tumor-DNA testing, which is used to provide information on the effectiveness of PARP inhibitors, and which could be used as a prescreen to germline testing. In this workflow, the DNA of the tumor was first tested for presence of PV in OC risk genes, which could be either somatic or derived from the germline. Only those patients with a tumor PV (15–20%) were referred to a clinical geneticist for germline testing [4, 5]. In the second workflow, the mainstreaming approach to genetic testing in OC, women were offered germline testing performed by the non-genetic healthcare professionals who were treating them during routine cancer care. These non-genetic healthcare professionals had received additional training to be able to provide genetic counseling [13–15]. Both alternative workflows improved the recognition of genetic predisposition in women with OC by increasing the percentage of (offering) genetic testing to a level exceeding 70% [4, 14].

The local implementation of these alternative workflows generated variations in the genetic-testing workflows for OC within the Netherlands. The presence of these variations called for national coordination in order to establish a uniform, high-quality genetic-testing workflow.

## Reaching national consensus on the preferred workflow: Tumor-First

To reach consensus on the preferred national genetic-testing workflow, leading professionals in the field organized multidisciplinary meetings to discuss the future of genetic testing for OC. These meetings were attended by patient representatives and healthcare professionals representing all disciplines involved in genetic testing for OC (gynecology, pathology, genetics, and medical oncology) from various regions of the Netherlands. Topics of discussion included the advantages and disadvantages of the regular genetic-testing workflow, as compared to the tumor-pre-testing approach and the mainstreaming approach. These meetings resulted in consensus on the future “Tumor-First” genetic-testing workflow for OC, which is based primarily on the tumor-DNA test as a pre-test for heredity. Once a tumor PV has been identified, the workflow includes both the option to refer the patient to a clinical geneticist and the option to use mainstream germline testing, if the gynecologists involved have been sufficiently trained to provide genetic counseling.

**Fig. 1:**
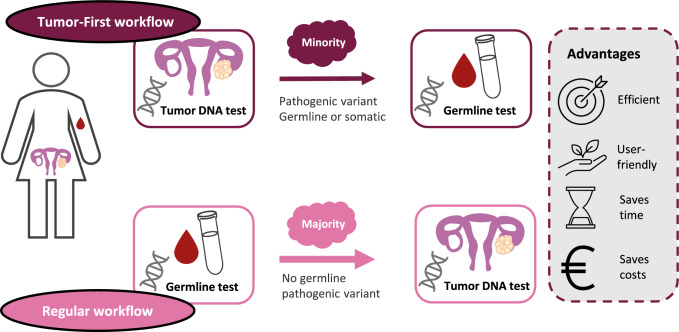
The Tumor-First workflow and the regular genetic-testing workflow (which starts with a germline test). The Tumor-First workflow is more efficient, as only patients with a tumor-pathogenic variant (i.e., a minority) receive a germline test. This is in contrast to the approach of starting with germline testing, in which all patients without a germline pathogenic variant (i.e., the majority) could be eligible for a tumor test in order to generate information on the effectiveness of PARP inhibitors. Note that guidelines regarding which patients are eligible for treatment with PARP inhibitors may evolve over time

The new Tumor-First workflow was created and selected for implementation on a nationwide scale, as it offers several advantages over the regular genetic-testing workflow, as visualized in Fig. 1. Using the tumor-DNA test as a pre-test for heredity efficiently stratifies the practice of clinical-genetics care. Only patients with a PV in tumor DNA (around 15–20%) receive an indication for extensive genetic counseling that may lead to germline testing [4, 5]. At the same time, the tumor DNA provides medical oncologists with information on the effectiveness of PARP inhibitors shortly after diagnosis and limits double testing (i.e., tumor testing after germline testing to detect somatic pathogenic variants). This workflow was therefore regarded as more efficient than other approaches. A cost-effectiveness analysis confirmed that the Tumor-First workflow reduced the average per-patient costs of genetic testing (i.e., including counseling, tumor, and germline testing) by 50% in an optimistic scenario of 100% testing uptake [16].

In addition, the Tumor-First workflow is less burdensome for most women, as more than 80% have no tumor PV and therefore no indication for germline testing [4, 5]. Moreover, given that all tumors are systematically subjected to genetic testing regardless of patient characteristics, the Tumor-First workflow has even been hypothesized to reduce existing social-demographic disparities in access to genetic testing [12, 17]. Implementation of the Tumor-First workflow seemed feasible in light of the success of local initiatives [4, 5].

## The Tumor-First workflow in more detail

### Multidisciplinary collaboration

**Fig. 2:**
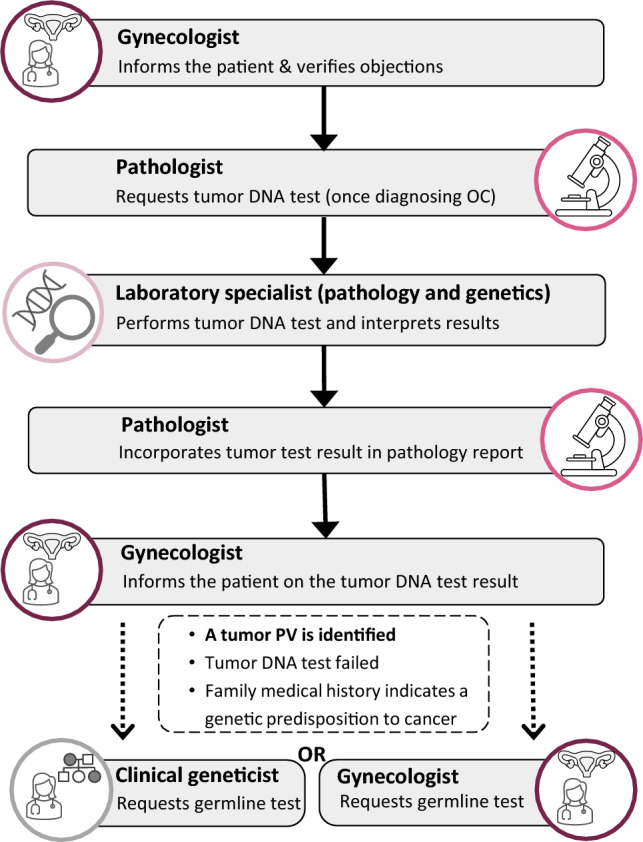
Tumor-First is a multidisciplinary workflow in which professionals from the disciplines of gynecology, pathology, and clinical genetics collaborate. Laboratory specialists in pathology are also known as clinical scientists in molecular pathology, and laboratory specialists in genetics are also known as clinical laboratory geneticists

The Tumor-First workflow for genetic testing in OC is a multidisciplinary workflow (Fig. 2). It starts when a patient with OC (or suspicion thereof) presents for a biopsy or surgery. All patients with OC are eligible for the Tumor-First workflow, regardless of OC grade or OC histology. A gynecological oncologist informs the patient about the Tumor-First workflow, checks for evidence of a genetic predisposition for cancer in the family, and ensures that the patient does not object to tumor-DNA testing as a prescreen to germline testing. In the rare event that a patient chooses to opt out, this is reported on the pathology request form. A pathologist then assesses the tissue and, if OC is diagnosed, requests the tumor-DNA test, if the patient has not opted out. The tumor-DNA test is performed in one of the eight hospitals with a center for clinical genetics. A laboratory specialist from the department of pathology (i.e., a clinical scientist in molecular pathology) assesses, interprets, and reports the results of the tumor-DNA test, in close collaboration with a laboratory specialist from the department of clinical genetics (i.e., a clinical laboratory geneticist). The results of the tumor-DNA test are then communicated to the gynecological oncologist, who informs the patient of these results and their consequences, in addition to entering the results into the patient’s electronic medical record. If a tumor PV is identified, if the tumor-DNA test has failed (or there was no tissue available), or if the family medical history indicates a genetic predisposition to cancer, the patient is eligible for extensive genetic counseling and, if wanted, germline testing. In that case, the gynecological oncologist either offers a referral to a clinical geneticist or (if sufficiently trained) offers counseling and germline testing through the mainstreaming approach. Referral to a clinical geneticist or mainstreaming can also be offered in cases where a tumor-DNA test was not possible or not wanted. The checklist for the Tumor-First workflow contains a detailed overview of the specific tasks for each discipline (Supplementary Table 1).

### Technical aspects and quality of the tumor-DNA test

The tumor-DNA tests for the Tumor-First workflow are performed on formalin-fixed, paraffin-embedded (FFPE) tumor material, originating either from resections, biopsies, or ascites. As the tumor-DNA test is used to select patients for germline testing, it is of utmost importance that the test does not miss pathogenic variants. The sensitivity must thus be similar to that of a germline test performed on DNA from leukocytes, which is possible as it concerns recently diagnosed OC with DNA of relatively good quality. The quality of the tumor-DNA test and the interpretation of the results must meet the national quality criteria and agreements for germline-DNA diagnostics. The tumor-DNA tests for the OC Tumor-First workflow are performed exclusively in non-commercial ISO 15189 accredited medical centers in which specialists from the departments of pathology and clinical genetics work in close collaboration. The validation and interpretation of the test are a joint responsibility of clinical scientists in molecular pathology and clinical laboratory geneticists, with consultation of pathologists and clinical geneticists. At a minimum, the tumor-DNA test must assess the genes that are at high risk of OC (i.e., *BRCA1* & *BRCA2*) and those that are at moderate risk of OC (i.e., *RAD51C*, *RAD51D*, *BRIP1*, and *PALB2*), as outlined in the national guideline on hereditary OC [3]. All centers use methods based on next-generation sequencing (NGS), combined with multiplex ligation-dependent probe amplification (MLPA) for *BRCA1*, if deemed necessary based on the NGS approach applied.

## The Tumor-First implementation project

**Fig. 3:**
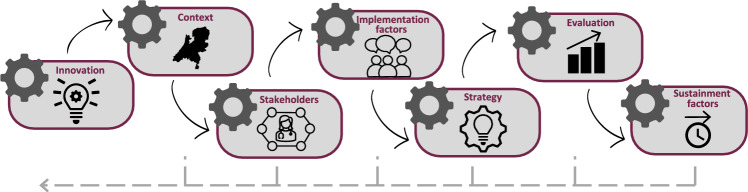
Framework for the implementation activities within the Tumor-First implementation project. The context and stakeholders were examined. Factors important to implementation were identified during focus-group discussions, and implementation strategies customized to these factors were selected. The implementation was evaluated, and factors important to sustainment were investigated. As indicated by the gray line, implementation is a continuous process that can involve new insights and adaptations

In 2020, a nationwide project was started with a multidisciplinary implementation team to implement the Tumor-First workflow. The activities within this project are visualized in Fig. 3. The implementation project commenced with an examination of the current situation (i.e., context and stakeholders), as has also been suggested as an initial step in the implementation of the change model developed by Grol and Wensing [18]. To identify factors that are important to implementation, multidisciplinary focus-group discussions were organized with healthcare professionals involved in the Tumor-First workflow for OC [19]. Using the framework developed by Flottorp et al., factors important for implementation were categorized and analyzed [20]. Healthcare professionals endorsed the nationwide implementation of the Tumor-First workflow, as it was regarded as an efficient workflow to detect heredity. To realize implementation on this scale, however, additional standardization and guidance was needed with regard to the quality of the tumor test and the logistics of the workflow. Another challenge involved communication between healthcare professionals (i.e., ensuring that all healthcare professionals were adequately informed on the progress and results of the Tumor-First workflow). In addition, healthcare professionals agreed on the importance of monitoring the inclusion of patients with OC. They further indicated that they would appreciate support in the implementation of the workflow within their daily work routines, as well as in monitoring the results of its implementation [19].

## Implementation strategies

We selected strategies tailored to the implementation factors for the Tumor-First workflow, as identified during the multidisciplinary focus-group discussions. Table 1 provides an overview of the multifaceted implementation plan, which consisted of five main elements. (1) We created awareness and increased knowledge among healthcare professionals by providing information and education; and (2) we developed practical tools to assist healthcare professionals in working according to the Tumor-First workflow. In addition, (3) we ensured that the Tumor-First workflow was embedded within national guidelines and guidance documents, and we (4) facilitated implementation by providing day-to-day support to healthcare professionals (i.e., project managers). Finally, (5) we monitored the effect of the implementation at the national level, and provided feedback to centers on their testing rates. Together, these implementation strategies played a central role within the implementation project. Several key activities are highlighted in Table 1.

**Table 1:** A tailored multifaceted implementation strategy for nationwide implementation of the Tumor-First workflow

Strategy	Example of activities	Engaging
Information/education	Information at the website National Tumor-First symposium (3 times) Outreach: conferences Outreach: articles and newsletters Educational video for clinicians	Team of professionals Patient organizations
Practical tools	Patient information letters Patient test-result letters Workflow checklist Smart phrases	Team of professionals Patient organizations
Embedding	National guideline for hereditary OC Embedding Tumor-First financially Guidance document for tumor-DNA testing	Team of professionals Healthcare financers Professional associations
Support and reminders	Annual multidisciplinary meetings for each center Daily contact and support Follow-up on steps not yet implemented	Team of professionals
Auditing and feedback	National monitoring of implementation Feedback results by region	Team of professionals

### Information/education and practical tools

A website was developed to contain extensive information on the Tumor-First workflow and the nationwide implementation project (www.tumorfirst.nl). This website also provides an online toolbox with practical tools and support for healthcare professionals (Supplementary Fig. 1). For example, the toolbox contains a step-by-step checklist of the tasks for healthcare professionals. It also contains educational videos for clinicians and letters for patients explaining the Tumor-First workflow and the tumor-DNA test results, all developed with the help of patient organizations. The toolkit further offers “smart phrases” that can help healthcare professionals to inform patients in a uniform manner and facilitate reporting in the electronic medical records of patients. The website served as a nationwide platform for sharing knowledge, tools, and news updates about the implementation project.

The Tumor-First workflow has been described in a number of scientific articles, and various national and international presentations on the project have been given at multidisciplinary conferences to create awareness. Each year, a Tumor-First symposium has been organized to facilitate the sharing of knowledge and best practices with all healthcare professionals involved.

### Embedding

The Tumor-First workflow has been embedded within the national guidelines for hereditary OC [3], and agreements have been made with regard to its financial embedding within the healthcare system [21]. In addition, a guidance document on Tumor-First testing was developed to enhance clarity on the prerequisites for the quality of a tumor-DNA test that can be used as a prescreen to germline testing, including example texts for reporting of the tumor DNA test result. This document was based on the results of an inventory conducted among pathologists, clinical scientists in molecular pathology, clinical laboratory geneticists, and clinical geneticists, as well as on recommendations from consensus meetings held among these professionals [22]**.**

### Support and reminders

Each year, center-specific multidisciplinary implementation meetings were organized in the centers where the tumor-DNA tests were being performed. A Tumor-First checklist was used during these meetings to evaluate which tasks were being performed according to the Tumor-First workflow and which tasks needed more attention. Tasks that had not yet been implemented were listed, and a plan of action was prepared and followed up through reminders. Project managers were available for day-to-day support. The amount and content of this support varied widely by region, due to organizational differences between regions and variations in level of experience with the Tumor-First workflow.

### Audit and feedback

The effect of the implementation was monitored over the years, and the testing rates within the Tumor-First workflow have been analyzed both nationally and regionally. Region-specific data compared to national data were provided as feedback at two timepoints during implementation. Regional meetings were organized to discuss these results with healthcare professionals and to evaluate what could be learned from the monitoring results. Two steps in the workflow proved critical for optimizing the detection of a genetic predisposition in OC: (i) ensuring that all OC patients receive tumor-DNA testing and (ii) ensuring that all patients with a tumor PV or a failed tumor-DNA test receive germline testing (or referral for such testing). Some centers had incorporated safety checkpoints (to avoid missing the inclusion of patients for tumor-DNA testing and/or germline testing) or monitored the implementation themselves at the regional level [23]. For example, some centers periodically prepared a list of patients with a tumor PV to check the follow-up. To facilitate monitoring in the future, we aimed to optimize registration in the electronic medical records and in national databases, including the Dutch Pathology Registry (PALGA) [24] and the Dutch Cancer Registry [25].

## Current status: Tumor-First successfully implemented nationwide

The journey from the initial conceptualization of this workflow to the nationwide implementation of these ideas was long and complex. The inception of the Tumor-First workflow can be traced back to as early as 2014/2015, when the initial ideas to use a tumor-DNA test as pretest to germline testing began to take shape. Since early 2023, all regions in the Netherlands have adopted the Tumor-First workflow for genetic testing in OC, and a national uniform workflow has been established. We regard the nationwide implementation of the Tumor-First workflow as successful, given that all regions currently have adopted the Tumor-First workflow as the standard genetic-testing practice. Moreover, healthcare professionals are positive, and testing rates are high. Although data collection to monitor the effect of the implementation is currently ongoing, the preliminary results seem promising. The percentage of OC patients receiving a tumor-DNA test has increased over the years, up to over 80% in 2021, as presented at the Ninth International Symposium on Hereditary Breast and Ovarian Cancer in 2023 [26]. Final results concerning the effects of the Tumor-First workflow implementation (i.e., testing rates from 2019 through 2023) are expected in 2024, along with the percentage of patients with a known germline status through the Tumor-First workflow.

## What did we learn?

Throughout the implementation of the Tumor-First workflow, we have learned valuable lessons that are potentially significant at a broader scale. First, multidisciplinary support from professionals involved in the workflow and patient organizations has formed the basis for this nationwide implementation, and mutual consensus on the preferred nationwide workflow has proven significant. Second, we learned that balancing standardization against allowing for regional variations is crucial in a nationwide implementation project. Standardization and a clear outline of the workflow are necessary to create a nationwide uniform workflow for genetic testing. At the same time, however, centers should be allowed to make their own interpretations of working according to the Tumor-First workflow, given the existence of regional differences in preferences and methods. Third, regional differences within the implementation of the Tumor-First workflow have been instructive. For example, we have learned that the implementation of the Tumor-First workflow is heavily reliant on collaboration between the departments of pathology and clinical genetics, which is more advanced in some regions than it is in others.

## The future

When considering the future, the following question arises: Will the results of our implementation be sustained after the project ends? This will be a focus of future research, in which we will aim to understand factors influencing the sustainability of our implementation results, thereby promoting a smooth transition from project-related implementation to sustainable daily clinical practice. Furthermore, in light of future developments within the field of clinical cancer genetics, there might be adaptations to the current diagnostic workflow. For example, in the near future, homologous recombination deficiency testing will be implemented within the workflow. Our nationwide network can help to minimize regional differences in the route and quality of genetic testing for this biomarker.

## Conclusion

This overview provides an example of how the successful implementation of a new workflow can create uniform, high-quality genetic testing at a nationwide level, thereby reducing regional differences in patient care and improving the genetic-testing rate of patients with OC. Based on the positive feedback we have received from the stakeholders, we conclude that our multifaceted implementation strategies have significantly contributed to the desired outcome of nationwide implementation. Our Tumor-First implementation project may serve as an example of how to approach the implementation of new genetic-testing workflows.

## Supplementary Information

Below is the link to the electronic supplementary material.

Supplementary file1 (PDF 311 kb)

## Data Availability

No datasets were generated or analysed during the current study.
