# Supplementary material for: Optimizing the detection of hereditary predisposition in women with epithelial ovarian cancer: nationwide implementation of the Tumor-First workflow
Source: Fam Cancer. 2024 May 29;23(4):429–36. doi: 10.1007/s10689-024-00398-9 (PMC11512879; doi:10.1007/s10689-024-00398-9)
Supplement: Supplementary file 1 — Supplementary file1 (PDF 311 kb) [file 10689_2024_398_MOESM1_ESM.pdf]

**Supplementary Tables and Figures**

Supplement to:

Witjes VM, Hermkens DMA, Swillens JEM, Smolders YHCM, Mourits MJE, Ausems MGEM, de Hullu JA, Ligtenberg MJL, Hoogerbrugge N. *Optimizing the detection of hereditary predisposition in women with epithelial ovarian cancer: Nationwide implementation of the Tumor-First workflow*

**Familial Cancer 2024**

**Supplementary Table 1:** Tumor-First checklist including all the tasks for gynecologists, pathologists, laboratory specialists, and clinical geneticists, as well as general areas of focus.

| **Tumor-First checklist** | |
|---|---|
| | |
| **Gynecology** | |
| | Briefly informs the patient about Tumor-First in OC, preferably prior to surgery |
| | Documents in the Electronic patient record (EPR) that the patient has been informed about Tumor-First and has not used the Opt-Out option (smartphrase) |
| | Hands out information letter about Tumor-First and/or refers to website, if needed |
| | Checks for evidence of hereditary predisposition to cancer in the family (ovarian, breast, endometrial, colorectal cancer in first or second degree) using the referral checklist and documents family medical history in EPR. |
| | Sends tumor tissue for standard pathology review (including request for Tumor-First test) |
| | Documents on the request form in case the patient objects to the Tumor-First test (Opt-Out) |
| **Pathology** | |
| | Receives tumor tissue from internal or external source |
| | Diagnoses ovarian/tuba carcinoma |
| | Includes all histological intra-ovarian or extra-ovarian carcinomas (no histological triage takes place) |
| | Revises (as part of the Tumor-First analysis), if necessary, tumor type on external applications (not obligatory) |
| | Verifies that the patient does not object to the Tumor-First analysis (Opt-Out) |
| | Checks the PALGA (Dutch pathology registry) database to determine whether any previous tumor DNA testing has been successfully completed |
| | Requests Tumor-First analysis |
| **Molecular pathology laboratory** | |
| | Receives the tumor tissue and the request for Tumor-First analysis |
| | Performs Tumor-First analysis with an assay that is validated in collaboration with a local clinical laboratory geneticist and clinical geneticist |
| | Interprets results of the Tumor-First analysis (collaboration between clinical scientist in molecular pathology and the clinical laboratory geneticist) and incorporates these results in the pathology report |
| **Pathology** | |
| | Incorporates the Tumor-First test result in the PALGA database |
| | Writes a clinical conclusion text that indicates whether the Tumor-First test was successful and whether the result is an indication for clinical genetic counseling (including family history as a reason for counseling) |
| | Approves PALGA report for linkage to EPR / or transmission to external requester |
| | Discusses Tumor-First results of patients from the hospital's own practice in the multidisciplinary team meetings (MDTs) |
| **Gynecology** | |
| | Receives the result of the Tumor-First test and discusses this with the patient |

| | |
|---|---|
| | Hands out the results letter from the Tumor-First test |
| | Refers the patient to clinical genetics, depending on test results and family history |
| | - If the Tumor-First test was not successful, the patient is routinely referred to clinical genetics |
| | Documents the results of the Tumor-First test in the EPR (NOTE: It is necessary to draw a clear distinction between tumor test and germline test results) |
| | Provides the medical oncologist and, if necessary, the peripheral gynecologist + general practitioner with the Tumor-First test results. |
| **Clinical genetics** | |
| | Informs patient about germline test and asks for consent |
| | Informs patient about the difference between the results of the tumor test and the germline test |
| | Requests germline test |
| | Informs patient about the results of the germline test and possible follow-up steps |
| | Records germline test results in the EPR (internal patients) |
| | Sends copy of the germline test result letter to the specialists involved |
| **General** | |
| | MDT between gynecology, molecular pathology laboratory, clinical genetics, pathology |
| | Communication plan for dissemination of the Tumor-First procedure within the region |
| | Working arrangements are included in the Standard Operation Procedures of the various departments |
| | Collaboration, authority, and responsibilities of the Pathology and Genetics departments on the Tumor-First test procedure and access to the required infrastructure are established within the quality systems of both departments |
| | Funding for the tumor test has been arranged |
| | Standard texts for reporting on Tumor-First analysis are available and have been approved by the departments of Pathology and Genetics |
| | Build in a check to periodically verify that the Tumor-First test has been requested |
| | Build in a safety net/check that patient has been referred to a clinical geneticist --> monitor request for germline test |
| | Validation report on the Tumor-First analysis used is present and accredited by a clinical scientist in molecular pathology and a clinical laboratory geneticist |
| | Tumor-First analysis is covered by ISO 15189 laboratory certification |

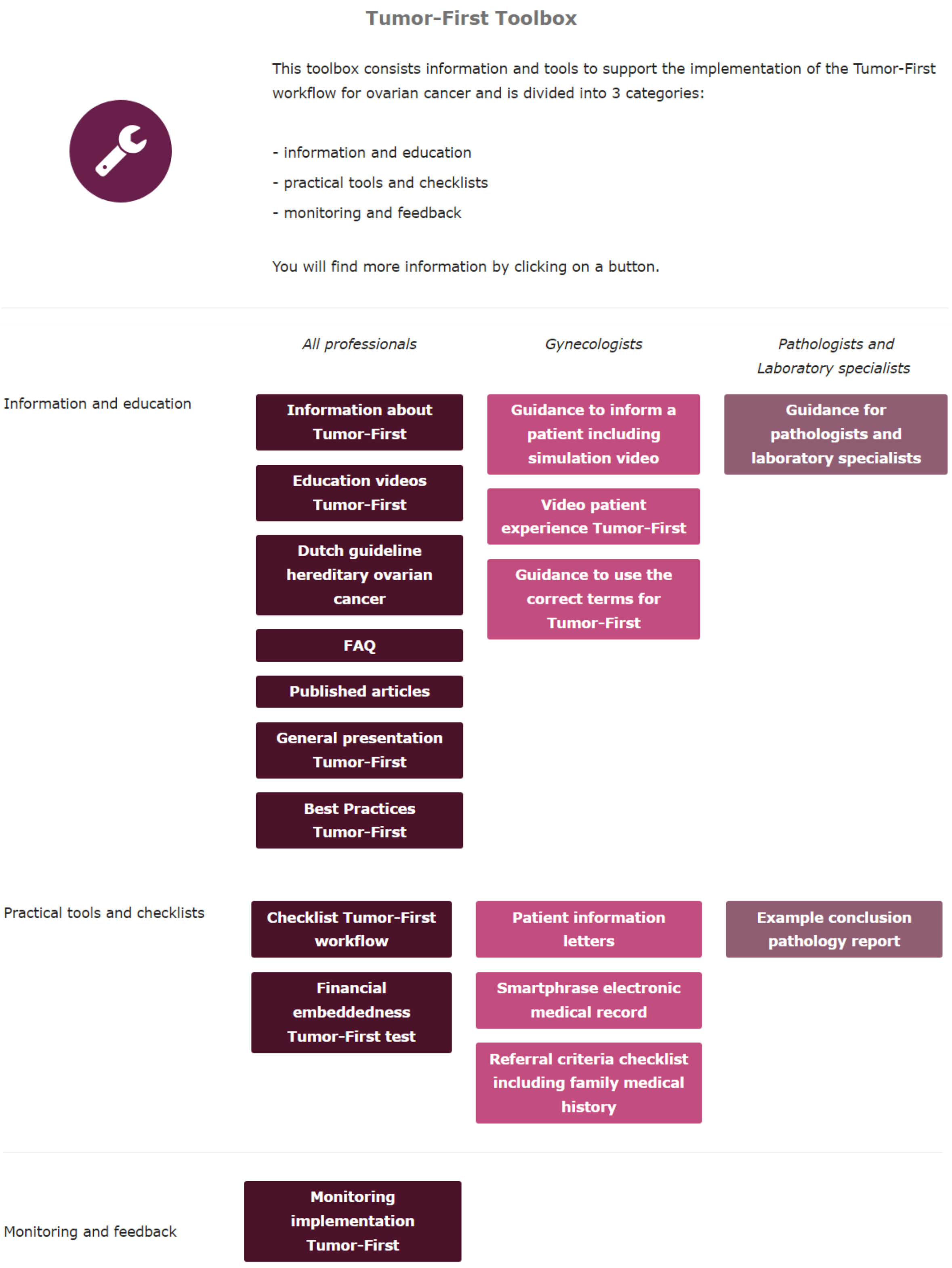


**Supplementary Figure 1**: A screenshot of the toolbox at the Tumor-First website. By clicking on a button in this toolbox, you can find more information and the tools. This is an English translation of the original toolbox in the Dutch language (www.tumorfirst.nl).
